# Gellan Gum/Pectin Beads Are Safe and Efficient for the Targeted Colonic Delivery of Resveratrol

**DOI:** 10.3390/polym10010050

**Published:** 2018-01-08

**Authors:** Fabíola Garavello Prezotti, Fernanda Isadora Boni, Natália Noronha Ferreira, Daniella de Souza e Silva, Sérgio Paulo Campana-Filho, Andreia Almeida, Teófilo Vasconcelos, Maria Palmira Daflon Gremião, Beatriz Stringhetti Ferreira Cury, Bruno Sarmento

**Affiliations:** 1 Faculdade de Ciências Farmacêuticas, Universidade Estadual Paulista (UNESP), Araraquara, Rodovia Araraquara–Jaú, Km 1, Araraquara 14801-902, Brazil; fabiolagp@gmail.com (F.G.P.); boni.fernanda@gmail.com (F.I.B.); noronhanat@fcfar.unesp.br (N.N.F.); pgremiao@fcfar.unesp.br (M.P.D.G.); curybsf@fcfar.unesp.br (B.S.F.C.); 2 Instituto de Química de São Carlos, Universidade de São Paulo (USP), Avenida Trabalhador São-Carlense, 400, São Carlos 13560-970, Brazil; danyss1986@gmail.com (D.d.S.e.S.); scampana@iqsc.usp.br (S.P.C.-F.); 3 I3S-Instituto de Investigação e Inovação em Saúde, Universidade do Porto, Rua Alfredo Allen, 208, 4200-135 Porto, Portugal; andreia.almeida@ineb.up.pt (A.A.); teofilo.vasconcelos@bial.com (T.V.); 4 INEB-Instituto de Engenharia Biomédica, Universidade do Porto, Rua Alfredo Allen, 208, 4200-135 Porto, Portugal; 5 CESPU-Instituto de Investigação e Formação Avançada em Ciências e Tecnologias da Saúde, Rua Central de Gandra 1317, 4585-116 Gandra, Portugal; 6 School of Pharmacy, Queen’s University Belfast, Medical Biology Centre, 97 Lisburn Road, Belfast BT9 7BL, UK

**Keywords:** gellan gum, pectin, resveratrol, mucoadhesive microspheres, cytotoxicity, in vitro permeability, Caco-2 cells, triple co-culture model

## Abstract

This work addresses the establishment and characterization of gellan gum:pectin (GG:P) biodegradable mucoadhesive beads intended for the colon-targeted delivery of resveratrol (RES). The impact of the polymer carrier system on the cytotoxicity and permeability of RES was evaluated. Beads of circular shape (circularity index of 0.81) with an average diameter of 914 μm, Span index of 0.29, and RES entrapment efficiency of 76% were developed. In vitro drug release demonstrated that beads were able to reduce release rates in gastric media and control release for up to 48 h at an intestinal pH of 6.8. Weibull’s model correlated better with release data and *b* parameter (0.79) indicated that the release process was driven by a combination of Fickian diffusion and Case II transport, indicating that both diffusion and swelling/polymer chains relaxation are processes that contribute equally to control drug release rates. Beads and isolated polymers were observed to be safe for Caco-2 and HT29-MTX intestinal cell lines. RES encapsulation into the beads allowed for an expressive reduction of drug permeation in an in vitro triple intestinal model. This feature, associated with low RES release rates in acidic media, can favor targeted drug delivery from the beads in the colon, a promising behavior to improve the local activity of RES.

## 1. Introduction

Gellan gum (GG) and pectin (P) are widespread available, low-cost, nontoxic, stable, biocompatible polysaccharides which exhibit gelling properties and have several structures able to be chemically and/or physically modified, providing promising characteristics for the design of drug delivery systems in the pharmaceutical field [1,2]. They have been also investigated as technological platforms due to their mucoadhesive properties—their ability to establish supramolecular interactions with mucosal components, such as mucin chains—which contribute to drug immobilization and retention in a target organ through more intimate contact over an extended period of time, favoring a local concentration gradient [3]. Additionally, while GG is degraded specifically in the colon by galactomannanas enzymes [4], P exhibits resistance to gastric and intestinal bacteria, being specifically fermented by *Bifidobacteria* and *Bacteroides* in the colon environment [5]. Since both polymers are degraded specifically by the colonic microbiota, it is noteworthy that they represent promising materials in the development of carrier systems to target drugs to the colon [1,5,6,7,8].

Mucoadhesion is a complex phenomenon and mucoadhesive ability can be related to the presence of reactive functional groups in the polymer chain capable of interacting with mucin present in the mucus [9]. Both GG and P are anionic polysaccharides with well-known mucoadhesiveness, related mainly to their numerous carboxyl groups that can interact through hydrogen bonds with mucin oligosaccharide chains [1,10,11,12,13,14,15,16].

Considering the ability of GG and P to form mucoadhesive microcapsules in the presence of cations such as Ca^2+^ and Al^3+^, ionically crosslinked beads based on GG or GG:P blends were previously designed by our research group, since mucoadhesiveness is an important property for multiparticulate systems intended for the colon-targeted delivery of drugs by the oral route [1,8]. The colon has a low mucus turnover rate and slow motility, which facilitates contact between polymeric systems and mucin glycoproteins and is responsible for the initial adhesive interaction that might involve chain interpenetration, prolonging residence time at the action site [1,8,17].

Blending P with GG was proposed in order to form a material with suitable properties in relation to the isolated polymers—improved mucoadhesiveness and mechanical properties—to allow for the effective control of drug release at different sites in the gastrointestinal tract (GIT) [8]. The control of drug release rates is based on a swellable polymer matrix that resists premature dissolution and/or degradation in the upper portions of the GIT at an acidic pH. The crosslinking process is a rational strategy to modulate such properties, and the correlation between the degree crosslinking of a polymer with the control of release rates of the entrapped drug has been demonstrated [1,8,18,19,20]. Additionally, effective drug targeting to the colon should be achieved since specific enzymatic degradation of the matrix should happen at this site by specific enzymes produced by the colonic microbiota [6,21].

The GG:P beads previously developed by Prezotti and co-authors (2014) were prepared by ionotropic gelation using AlCl_3_ as a crosslinking agent, and their high mucoadhesive ability was evidenced by in vitro and ex vivo experiments. In addition, the beads were able to reduce release rates of the loaded drug (ketoprofen) in acidic media (pH 1.2) and prolong drug release in phosphate buffer (pH 7.4) for up to 6 h. The advantageous features of these beads reveal that they are a promising carrier for protecting drugs and targeting them to specific sites in the GIT in order to treat local diseases or modulate drug permeability [8].

RES has attracted great attention due to its wide range of beneficial effects in humans and several drug delivery systems have been proposed to carry this drug. It has been recognized as a promising compound for the treatment of several illnesses, such as metabolic and cardiovascular diseases, neurodegeneration, and ischemic injuries, and for both the prevention and treatment of several types of cancer [22,23,24,25].

Resveratrol (RES), a class II in the Biopharmaceutical Classification System, is a natural polyphenol with recognized antioxidant properties found in grapes, red wine, peanuts, mulberries, and some medicinal plants [26], and is potentially useful as a dietary supplement or for use in natural medicine [22,23]. However, the labile properties, low solubility, and rapid and extensive metabolism of RES impose great challenges for its applications in therapeutics, mainly, by the oral route [25,27].

Such features make the amount of RES that reaches the colon following oral administration insufficient to promote the desired beneficial effects at the target site, making a carrier system necessary for the effective delivery of RES to the colon. Additionally, the high permeability of RES opposes the accumulation of the drug in the colon [28].

The in vitro intestinal permeability of RES was evaluated using an innovative triple co-culture model consisting of Caco-2 cells, to reproduce the characteristics of the enterocytes; HT29-MTX cells, capable of secreting mucus; and Raji-B cells, which induce differentiation of Caco-2 cells into M cells [29,30,31]. Co-culture models that use multiple cell lines together enable accurate reproduction of an organ of interest, as is the case in this triple co-culture which consists of three different cell lines, exactly mimicking the human intestinal epithelium [30], being more physiological, functional, and reproducible for the study of drug permeability [29].

In this study, we showed that crosslinked mucoadhesive GG:P beads are a rational strategy to protect RES from the harsh environment of the stomach and modulate its release rates and biological interactions, and is an efficient and safe system for the targeted colonic delivery of this promising drug by the oral route.

## 2. Materials and Methods

### 2.1. Chemicals

Pectin (type LM-5206 CS) and gellan gum (Kelcogel^®^ CG-LA) were kindly provided by CP Kelco (Limeira, Brazil). Resveratrol was purchased from Sigma-Aldrich (São Paulo, Brazil). Transwell^®^ cell culture inserts were from Corning (Madrid, Spain). Dulbecco’s Modified Eagle’s Medium (DMEM) was from Lonza (Basel, Switzerland). Non-essential amino acids and fetal bovine serum were purchased from Biochrom GmbH (Berlin, Germany). Hank’s Balanced Salt Solution (HBSS) was from Gibco (Invitrogen Corporation, Life Technologies, Paisley, UK). Phosphate Buffered Saline and 3-(4,5-dimethyl-2-thiazolyl)-2,5-diphenyl-2H-tetrazolium bromide (MTT reagent) were from Sigma-Aldrich (Sintra, Portugal). Triton^®^ X-100 was from Merck (Oeiras, Portugal). All other materials used were of analytical grade and obtained from commercial suppliers.

### 2.2. Bead Preparation

Beads were prepared as previously described [8]. Briefly, aqueous dispersions of GG:P (4:1) at a concentration of 2% (*w*/*v*) were prepared under magnetic stirring at 80 °C until complete homogenization. RES was added to the dispersion at 0.5% (*w*/*v*) at 40 °C and kept under magnetic stirring for an additional 15 min. The dispersions were dropped through flat-tipped needles (23 G, 25 × 0.6 mm) into a cooled solution (8 °C) of AlCl_3_ (3%, *w*/*v*) under magnetic stirring (60 rpm). The crosslinking reaction was kept for an additional 45 min in an ice bath for strengthening of the beads. After, beads were separated by filtration, washed with distilled water to remove unreacted crosslinking agent, and dried at room temperature. Beads were prepared in duplicate and were stored in a desiccator containing silica gel.

### 2.3. Bead Characterization

#### 2.3.1. Particle Size, Size Distribution, and Morphology

Images of RES-loaded beads were taken on a Leica MZ APO^®^ stereoscope (Leica Microsystems, Wetzlar, Germany) coupled to the Motic Images Advance 2.0^®^ program. Fifty beads, previously dried until constant weight, were used to determine the average particle size, size distribution (Span index), and average circularity [1,8]. Span index was determined using the following Equation (1):

Span = (*D*_90_ − *D*_10_)/*D*_50_ (1)

where *D*_90_, *D*_10_, and *D*_50_ are the diameters (μm) determined for the 90th, 10th, and 50th percentiles, respectively.

#### 2.3.2. Attenuated Total Reflectance-Fourier Transform Infrared (ATR-FTIR) Spectroscopy

Infrared spectroscopy of GG, P, RES, empty beads, and RES-loaded beads was performed using a Vertex 70 Fourier Transform Infrared (FTIR) spectrometer (Bruker Optics Inc., Billerica, MA, USA) equipped with a Golden Gate single reflection ATR accessory and DLaTGS detector to investigate polymer–drug interactions [32,33]. Powdered samples were scanned over a wave region of 400–4000 cm^−1^.

#### 2.3.3. Entrapment Efficiency (%EE)

Immediately after being prepared, eight beads were grounded with 20 mL of phosphate buffer (pH 7.4) in a centrifuge tube using a high-shear homogenizer (Ultra-turrax IKA^®^, Campinas, Brazil) at 11,000 rpm for 2 min in order to extract the encapsulated drug. Sodium lauryl sulphate (1%, *w*/*v*) was added to ensure total drug solubilization. After 1 h, the dispersions were centrifuged to remove polymeric debris (2500 rpm; 5 min) and the amount of RES in the supernatant was quantified using a Cary 60 UV-Vis spectrophotometer (Agilent Technologies, Santa Clara, CA, USA) at 320 nm, in five replicates. The measured drug loaded (%DL_m_), theoretical drug loaded (%DL_t_), and entrapment efficiency (%EE) were calculated according to Equations (2)–(4), respectively [34]:

%DL_m_ = (weight_drug in beads_/weight_beads_) × 100 (2)

%DL_t_ = (weight_added drug_/weight_polymers+drug_) × 100 (3)

%EE = (%DL_m_/%DL_t_) × 100 (4)

where weight_drug in beads_ (μg) is the amount of RES in eight beads and weight_beads_ is the average mass (μg) of eight dried beads determined after drying the batches until constant weight. The %DL_t_ was determined based on bead composition, where weight_added drug_ is the mass (μg) of RES added during bead preparation, and weight_polymers+drug_ is the sum of the masses (μg) of GG, P, and RES added during bead preparation.

### 2.4. Drug Release Studies

In vitro drug release was performed using an SR8-Plus Hanson Dissolution Test Station (Chastworth, CA, USA) equipped with USP apparatus 1 (basket) at 50 rpm and 37 ± 0.5 °C. Solutions with different pH values were used to simulate the variations in pH along the GIT according to previously published methodology with minor modifications [1,8]. Initially, dissolution was carried out in acid media (0.1N HCl pH 1.2) containing sodium lauryl sulfate (1%, *w*/*v*) as surfactant over 2 h. Posteriorly, 0.2 M of tribasic sodium phosphate was added to achieve a pH of 6.8 (enteric media). Aliquots of 2 mL were withdrawn at pre-established intervals and immediately replaced with the same volume of fresh dissolution media at 37 ± 0.5 °C. The amount of RES released was quantified on a Cary 60 UV-Vis spectrophotometer (Agilent Technologies, Santa Clara, CA, USA) at 320 nm. Tests were performed in triplicate, using a mass of beads corresponding to 5 mg of RES and the same amount of free RES.

#### Kinetics of Drug Release

RES release data were fitted to different mathematical models (Korsmeyer-Peppas, Higuchi, First-order, Weibull, Hixson-Crowell, and Baker-Lonsdale) to evaluate the mechanism of drug release from the beads.

### 2.5. Cell Lines

The Caco-2 (C2BBe1) cell line (passages 64–66) was obtained from the American Type Culture Collection (ATCC, Manassas, VA, USA). Mucus-secreting HT29-MTX (passages 38–40) and Raji-B cell lines were kindly provided by Dr. T. Lesuffleur (INSERM U178, Villejuif, France) and Dr. Alexandre Carmo (Cellular and Molecular Biology Institute (IBMC), Porto, Portugal), respectively. Cells were cultured in plastic cell culture flasks (75 cm^3^) using Dulbecco’s Modified Eagle’s Medium (DMEM) supplemented with 10% fetal bovine serum (FBS), 100 U/mL penicillin, 100 mg/mL streptomycin, and 1% non-essential amino acids, and incubated in a humidified atmosphere with 5% CO_2_ at 37 °C using a conventional incubator (Binder^®^, Tuttlingen, Germany). Adherent cells (Caco-2 and HT29-MTX) were harvested at a confluence of 70–80%, using trypsin to detach them, and seeded in new flasks. The medium was changed every third day. Raji-B cells were grown in suspension cultures and had their medium changed when necessary.

### 2.6. Cell Viability

Cell viability was assessed by methyl-thiazolyl-tetrazolium (MTT) assay which allows for measurement of the percentage of viable cells due to living cells having to metabolize the MTT reagent into a colored, reduced product: purple formazan crystals [35]. The cytotoxicity of polymers, free RES, RES-loaded beads, and empty beads was studied in Caco-2 and HT29-MTX cell lines.

Cells were seeded at a density of 0.12 × 10^6^ cells/well in 24-well plates using supplemented DMEM. After 24 h incubation in a Binder^®^ incubator at 37 °C with a humidified atmosphere and 5% CO_2_, the medium was removed, the wells were washed with phosphate buffered saline solution (PBS) at 37 °C, and cells were incubated in the presence of isolated polymers (GG or P; 500–2500 µg/mL), empty and RES-loaded beads (suspended in HBSS—6, 8 and 10 beads/well) and free RES (diluted in HBSS) in concentrations corresponding to the amount of RES loaded in the beads (350, 450 and 600 µg/mL). The positive control (100% cell viability) was obtained by incubating them in the presence of HBSS or HBSS containing 0.5% DMSO (as found in free-RES stock solution). The negative control (cell death) was obtained by incubating cells in the presence of Triton^®^ X-100 (1% *w*/*v* in PBS). Cells were incubated under the same conditions described above for 24 h in the presence of the polymers and 4 h in the presence of beads and free RES. After this period, the wells were washed twice with PBS at 37 °C to remove the drug and the beads, and 1 mL of the MTT reagent (0.5 mg/mL in PBS) was added to each well. Plates were incubated for an additional 4 h at 37 °C. Subsequently, the content of the wells was carefully removed and 1 mL of dimethyl sulfoxide (DMSO) was added to each well in order to solubilize the formazan crystals originated by living cells. Plates were allowed a period of 10 min of continuous stirring inside a Synergy 2^®^ microplate reader (Biotek Instruments Inc., Winooski, VT, USA) before reading the absorbance at 590 nm (test wavelength) and 630 nm (background wavelength). Tests were performed in at least two independent experiments, each performed in quadruplicate.

### 2.7. In Vitro Intestinal Permeability

Permeability experiments were performed on a triple co-culture cell model. Co-cultures of Caco-2 and HT29-MTX at a 90:10 ratio were seeded in Transwell^®^ inserts of 24 mm diameter, with a translucent permeable membrane of polycarbonate, pore size of 3 μm, and a growth area of 4.67 cm^2^, at a density of 1.0 × 10^5^ cells/cm^2^ to a final volume of 1.5 mL (the apical compartment) in each insert. The inserts were placed in 6-well plates and 2.5 mL of supplemented DMEM was added to the basolateral compartment. The plates were maintained inside a Binder^®^ incubator at 37 °C with a humidified atmosphere and 5% CO_2_ [29,30]. Then, after 14 days, 1.0 × 10^6^ Raji-B cells was added to the basolateral chamber. After the addition of these cells, the media in this compartment was kept without changes until the permeability tests on the 21st day.

To perform the in vitro permeability experiments, media was carefully removed from the apical and the basolateral compartments; the inserts and wells were gently washed twice with PBS (pH 7.4) at 37 °C to remove all supplemented DMEM, filled with HBSS (1.5 and 2.5 mL, respectively), and allowed to equilibrate for 30 min. Afterwards, the media from the apical compartment was removed and 1.5 mL of free RES at 50 μg/mL in HBSS was added. The beads were placed directly in the apical compartment without removing the media. Plates were placed inside an orbital shaking incubator (IKA^®^ KS 4000 IC, IKA, Staufen, Germany) at 100 rpm and 37 °C. Aliquots (200 μL) were withdrawn from the basolateral chamber at predetermined times (5, 15, 30, 45, 60, 90, 120, and 180 min) and immediately replaced with HBSS. At the end, an aliquot from the apical compartment was collected [36]. Tests were performed in triplicate and an insert without the addition of sample was used as a control.

Before, during, and at the end of the permeability experiments, the Transepithelial Electrical Resistance (TEER) was measured using an EVOM2^®^ epithelial voltohmmeter with chopstick electrodes (World Precision Instruments, Sarasota, FL, USA) in order to monitor the formation, confluence, and integrity of the cell monolayers. Experiments were performed in triplicate.

The concentration of RES in the samples was determined by high-performance liquid chromatography (HPLC) analysis using a Waters HPLC system and data was processed with Empower 3^®^ Software (Waters Corporation, Milford, MA, USA). The stationary phase consisted of a C18 reversed-phase column Waters Symmetry Shield RP18 (3.5 μm, 100 × 4.6 mm) at 30 °C. The mobile phase consisted of (A) water and (B) acetonitrile (65:35, *v*/*v*) in isocratic mode and a flow rate of 1 mL/min. The run time was set at 10 min, the injection volume used was 50 μL, and the detection by UV was fixed at 307 nm.

The drug apparent permeability (*P*_app_) was calculated from the following Equation (5):

*P*_app_ = [(d*Q*/d*t*) × *V*]/(*A* × *C*_0_) (5)

where *P*_app_ is the apparent permeability (cm/s); d*Q*/d*t* (μM/s) is the flux across the monolayer obtained from the angular coefficient of the curve of the amount of drug transported versus time; *V* (cm^3^) is the acceptor chamber volume, which in this case corresponds to 2.5 cm^3^ (basolateral chamber); *A* (cm^2^) is the insert membrane growth area (equal to 4.67 cm^2^ for a 6 well plate); and *C*_0_ (μM) is the initial concentration in the apical compartment [37,38].

### 2.8. Statistical Analysis

Experiments were performed in triplicate and represented as mean ± standard deviation (SD). Statistical significance between different treatments was determined using analysis of variance (ANOVA) and post hoc Tukey’s test. Cytotoxicity results were analyzed by two-way analysis of variance (ANOVA) with Bonferroni Multiple/Post Hoc Group Comparisons (GraphPadPrism Software Inc., La Jolla, CA, USA). A 5% level of significance was adopted.

## 3. Results and Discussion

### 3.1. Bead Characterization

Beads were prepared by an ionotropic gelation technique, by dripping the negatively-charged polymer dispersion containing RES into a crosslinking solution containing the positively-charged aluminum ions. Beads are formed as the droplets enter the crosslinking solution, and are strengthened due to the crosslinking reaction between the polyanions (GG and P) and the trivalent ions (Al^3+^) [8]. Beads presented an average diameter of 913.70 ± 102.73 μm and a unimodal size distribution with a Span index of 0.29, indicating low polydispersity. The high circularity index values (0.81 ± 0.12) demonstrated the circular shape of the beads. Photomicrographs of GG:P beads evidencing their spherical morfology can be seen in the previous work from our research group [8].

**Figure 1:**
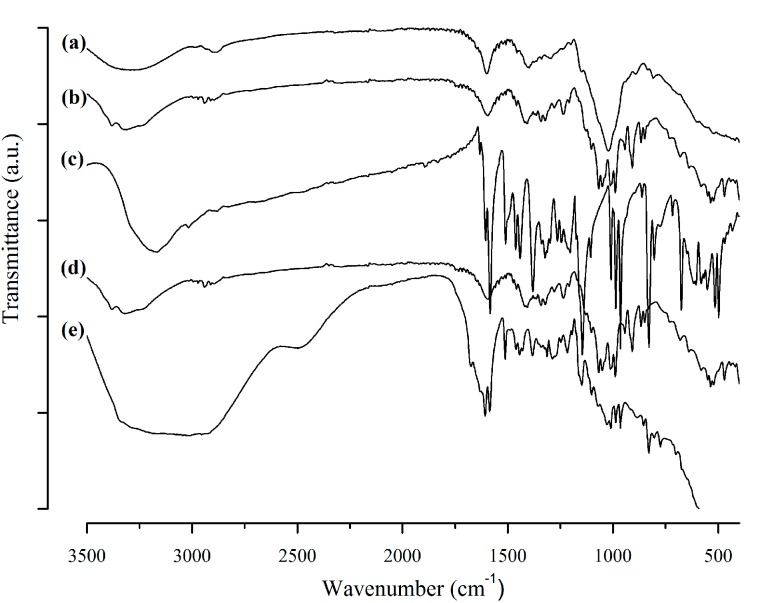
ATR-FTIR spectra of (**a**) gellan gum, (**b**) pectin, (**c**) resveratrol, (**d**) empty beads, and (**e**) RES-loaded beads.

The chemical interactions between bead components and RES were investigated by analyzing peak variation of GG, P, RES, empty bead, and RES-loaded bead spectra using ATR-FTIR (Figure 1).

GG spectra exhibited a broad band between 3400–3100 cm^−1^ due to O–H stretch vibrations of hydroxyl groups; C–H stretch vibrations of –CH_2_ groups were recorded at around 2925 cm^−1^, asymmetric and symmetric carboxylate anions were recorded stretching at 1600 and 1400 cm^−1^, respectively, while C–O stretching occurred at 1033 cm^−1^ [8,39]. Pectin showed a broad band related to O–H stretching vibrations between 3400–3100 cm^−1^. Characteristic C=O stretching of COOH groups close to 1700 cm^−1^ and asymmetric and symmetric carboxylate anion stretching resulted in a profile similar to that presented by GG [40]. The fingerprint region of pectin spectra comprises the pyranose cycle vibrations region with five characteristic bands around 1000 cm^−1^ [41]. Characteristic peaks of RES can be observed at around 990 cm^−1^ related to the trans-olefinic band, at 1380 cm^−1^ related to C–O stretching, and at 1580 cm^−1^ related to C–C olefinic stretching. A striking sign at 1600 cm^−1^ refers to the aromatic double band stretching [33]. Empty beads exhibited a profile similar to GG and P spectra, while RES-loaded beads presented characteristics peaks of RES. These data highlight the chemical stability of the drug inside the beads and the absence of chemical interactions between polymers and RES, suggesting that it might be physically entrapped within the polymer chains.

Beads presented a %EE of 75.7% ± 0.8%. The high drug loading may be related to the low solubility of RES in aqueous solutions that hindered drug diffusion to the crosslinking media during beads preparation. Similar results were achieved with other systems based on GG and blends of GG:P using AlCl_3_ as a crosslinking agent and containing ketoprofen [1,8].

### 3.2. Drug Release Studies

The main challenge in targeting drugs to lower segments of the GIT relates to the great variations in pH and enzymatic content that the system will face in its transit along different organs until reaching the colon. Subsequently, a successful colon-specific drug delivery system should protect the encapsulated drug against the low pH and enzymes of the stomach, preventing premature drug release in this organ, and should release it when reaching a higher pH, such as that of the colonic environment [42].

**Figure 2:**
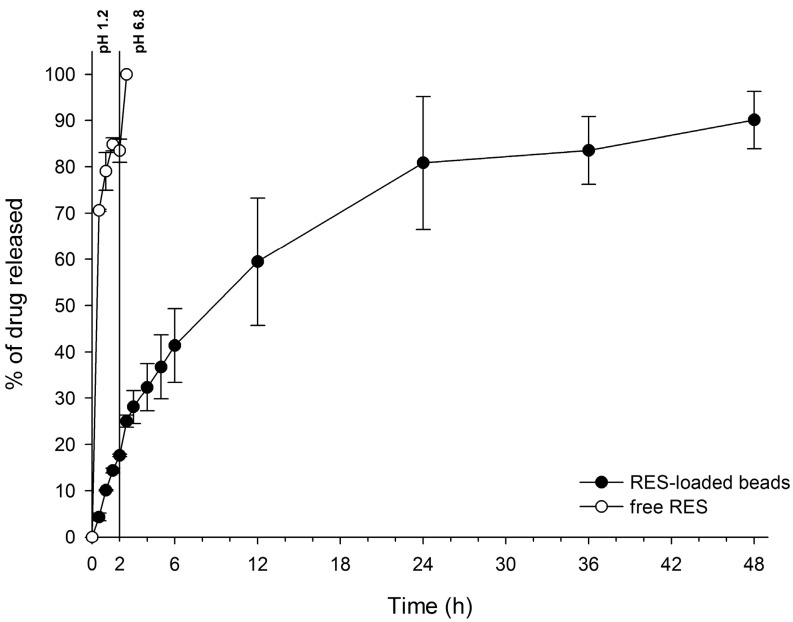
Dissolution profile of RES (free and loaded in GG:P beads) in media that mimic gastric (1.2) and enteric pH (6.8) (*n* = 3; mean ± SD).

Beads were able to significantly reduce release rates in acid media compared to the free drug. From the in vitro release profile of free RES and RES-loaded beads (Figure 2) it is possible to observe that only 17.6% ± 0.3% of the drug was released from the beads in acidic media (pH 1.2) after 120 min of experiment, while 83.5% ± 2.5% of free RES was already dissolved at the same time. Free RES completed its dissolution in only 150 min, right after changing the pH to 6.8. However, GG:P beads were able to control drug release for up to 48 h without any burst effect.

Weibull’s mathematical model fitted better with drug release data (*r*^2^ = 0.998). For this model, the *b* parameter can be used to indicate the mechanism of drug transport throughout the polymer matrix. Values of *b* ≤ 0.75 are related to release by Fickian diffusion, while values of 0.75 < *b* < 1 correspond to a combination of Fickian diffusion and Case II transport. When *b* > 1, drug transport follows a complex release mechanism. The value of the *b* parameter obtained was 0.79, indicating that the release mechanism of RES from the bead matrix was governed by both Fickian diffusion, in which drug release occurs due to a concentration gradient between the polymeric matrix and the dissolution media, and Case II transport, which is related to matrix swelling [19,43,44]. Thus, it can be concluded that both diffusion and swelling/polymer chains relaxation are processes that contribute equally to the control of drug release rates, i.e., the rates of chains relaxation and drug diffusion through the swelled matrix must be similar and are limiting to the release process [19,45].

### 3.3. Cell Viability

The in vitro cytotoxicity and cell permeability of crosslinked mucoadhesive GG:P beads were analyzed in order to assure safety of oral administration and evaluate the potential for modulating interactions with the cells; a feature favoring the local treatment of colonic disorders such as ulcerative colitis and colonic cancer. Cytotoxicity was evaluated using cell lines from the human intestine (Caco-2 and HT29-MTX).

In vitro cell viability assays are extremely important in the development phase of new drug delivery systems once they can predict the biocompatibility and the cytotoxicity. The colorimetric MTT assay is one of the most used methods for screening of cytotoxicity. Cells with intact metabolic activity are able to reduce the MTT reagent (3-(4,5-dimethylthiazol-2-yl)-2,5-diphenyl tetrazolium bromide), originating a purple formazan product that precipitates and it is then solubilized with an organic solvent—usually DMSO. This assay can, therefore, by the amount of purple product formed, determine cell viability and infer the harmful intracellular effects of the systems on cell metabolic activity [35,46], indicating drug carrier toxicity. A threshold of 70% cell viability was considered as the toxic level according to the ISO 10993-5 guideline [47].

**Figure 3:**
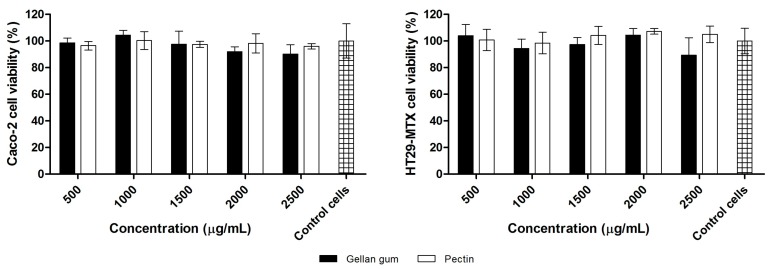
Cell viability (%) of Caco-2 and HT29-MTX cell lines after 24 h of incubation with increasing concentrations (500–2500 μg/mL) of GG and P (mean ± SD).

Analyzing Figure 3, it is possible to observe that both polymers used to prepare the beads did not decrease cell viability after 24 h of incubation for both cell lines. After incubation with GG, HT29-MTX and Caco-2 cell viabilities were found to be higher than 89.4% and 90.2%, respectively, for all concentrations. Similar behavior was found for P, in which the percentage of viable cells for the HT29-MTX cell line was higher than 98.5%, and higher than 96.0% for Caco-2 cells. The increase in GG concentration did not significantly affect HT29-MTX or Caco-2 cell viability (*p* > 0.05). The same was observed for P, since no significant differences were observed in cell viability among the different polymer concentrations (*p* > 0.05). These results show that, within the evaluated concentrations, GG and P are nontoxic polymers to Caco-2 and HT29-MTX cell lines.

**Figure 4:**
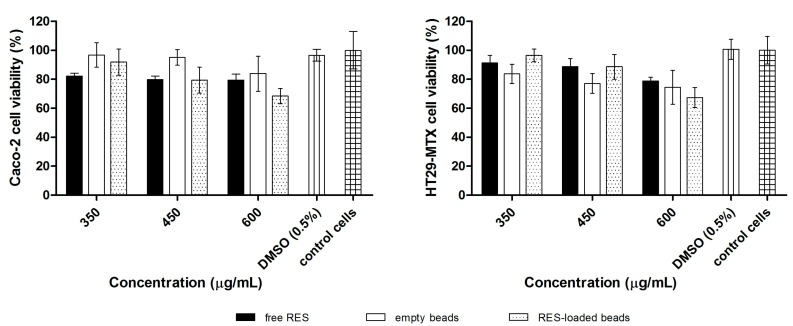
Cell viability (%) of Caco-2 and HT29-MTX cell lines after 4 h of incubation with increasing concentrations of free RES, and empty and RES-loaded beads (mean ± SD).

Beads biocompatibility, and therefore their safety to act as drug carriers for the delivery of RES to the colon, was evaluated by cell viability experiments. The percentage of viable cells from the different cell lines treated with empty beads, RES-loaded beads, and free RES, after 4 h of incubation with increasing concentrations of samples, is shown in Figure 4.

No significant cytotoxicity was observed when Caco-2 or HT29-MTX cell lines were incubated with HBSS containing 0.5% DMSO, indicating that DMSO up to 0.5% (*v*/*v*) can be used to prepare RES solutions for cytotoxicity studies. It was also observed that free RES and empty beads did not present a cytotoxic effect within the evaluated concentrations for both cell lines.

RES-loaded beads in concentrations of 6 and 8 beads/well did not reduce cell viability, however, at the highest concentration (10 beads/well) a discrete cytotoxic effect was observed, with 67.40% and 68.44% of cell viability for HT29-MTX and Caco-2 cell lines, respectively.

Comparing empty with RES-loaded beads reveals that the encapsulated RES did not influence the cell viability of both cell lines after 4 h of incubation (*p* > 0.05).

The cells were incubated in the presence of increasing concentrations of free RES (350–600 μg/mL), equivalent to the total amount of drug encapsulated in the beads (6 to 10 beads/well). The cell viability of HT29-MTX and Caco-2 cell lines after 4 h in the presence of RES-loaded beads was equal to that of free RES in all tested concentrations (*p* > 0.05).

Moreover, it is noteworthy that this assay was performed to evaluate the oral safety of the materials and to select non-toxic concentrations for the in vitro permeability experiments using the same cell lines, once the concentration of drug or drug carrier cannot present toxicity towards the cells that form the monolayer in the insert. Altogether, from the cell viability experiments, it can be concluded that GG:P beads are safe towards HT29-MTX and Caco-2 cells.

### 3.4. In Vitro Intestinal Permeability

The use of cell-based in vitro models allows the evaluation of drug permeability in conditions close to in vivo. These studies are of fundamental importance in the early stages of new drug delivery system development to ensure both success and reproducibility. Additionally, in vitro techniques avoid the use of laboratory animals and are less laborious and more economic than in vivo experiments [29].

The triple co-culture model used in this work has been recently established and optimized, gaining attraction as a more reliable model that more closely resembles intestinal mucosal architecture and, thus, the in vivo conditions found in humans to study drug permeability. It also allows evaluation of the effects of mucus on drug transport, and, therefore, it is a useful tool in studies with mucoadhesive systems [29,30].

**Figure 5:**
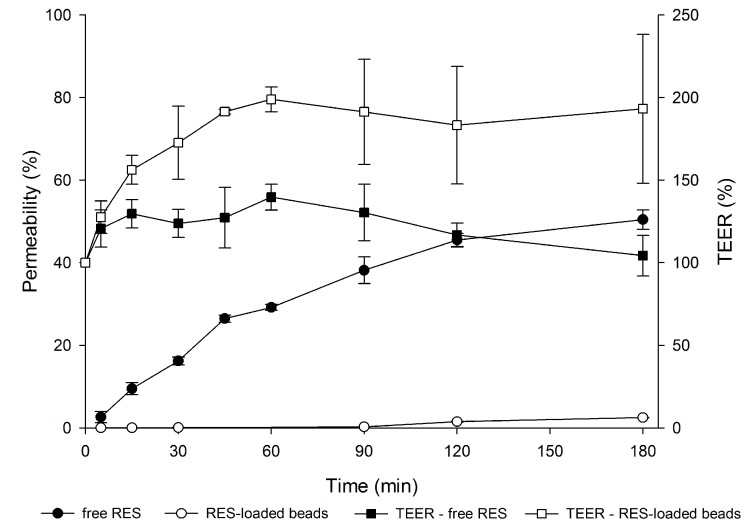
Permeability profile of free RES and RES-loaded beads across a triple co-culture model and TEER (%) of the cell monolayer as a function of time during the experiment (*n* = 3; mean ± SD).

In order to perform the in vitro permeability experiments, non-toxic concentrations of free RES and RES-loaded beads were selected based on cell viability results (Section 3.3). Tests were carried out uni-directionally from the apical to the basolateral compartment. The drug permeability profile is shown in Figure 5.

Regarding RES permeability data (Figure 5), it is evident that there is a significant reduction in the permeability of RES carried in mucoadhesive beads compared to that of RES solution, which reached 50.4% permeation versus 2.49% for the encapsulated drug (*p* < 0.05).

The high *P*_app_ value of free RES (14.2 × 10^−6^ cm/s), 16 times higher than that of the encapsulated drug (0.87 × 10^−6^ cm/s), evidences the ability of GG:P beads to modulate the permeation of the drug. In fact, the high permeability of free RES is in agreement with previous studies on RES permeability through Caco-2 cells [48].

The significant impact of the bead carrier on the permeability of RES is a relevant and promising feature that may favor the accumulation of the RES in the colon. Regarding this behavior, important concerns must be considered. Firstly, the high permeability of free RES through this cell model indicates that the mucus layer that covers the cell in the triple co-culture model did not impose great resistance against the diffusion of RES molecules.

From this appointment, it may be possible to suppose that the retention of beads on the outer side of the mucus layer and the low drug release rates even at pH 6.8 (Section 3.2), near to 7.4 of this test, may be imperative for the low permeability of RES. Additionally, the mucoadhesiveness of the GG:P beads [8] may improve the interaction of RES with cells, prolonging the contact with the colonic cells, which might improve local activity at this site.

In addition, it was observed that for free RES, a decrease in TEER percentage occurred during the permeability test (Figure 5). It has been described in the literature that the opening of the tight junctions leads to a reduction in TEER. However, a minor damage in the monolayer is also able to drastically reduce the values of TEER. According to Odijk and co-authors, a change of around 0.4% in the area of cell coverage inside the insert could reduce TEER by 80% [49]. According to these authors, TEER values can be lower due to damage or small gaps, but the monolayer can still show good barrier function with strong tight junctions as demonstrated by fluorescent staining [49].

In contrast, for the encapsulated drug, increased TEER values (%) were observed during the experiment (from ~150% to 250%). This behavior may be related to the deposition of polymeric debris due to bead erosion on the cell layer, since significant erosion of these beads (~25%) after 2 h at a pH of 7.4 was previously reported [8].

Hence, observing the final TEER percentages of the triple co-culture model after contact with free RES and the encapsulated drug, it can be concluded that the monolayers remained relatively intact at the end of the experiments and that the variations in TEER values might not be related to cell death. In this way, the permeability results really reflect the drug transport across the cell monolayers.

## 4. Conclusions

In this work, RES was successfully encapsulated in mucoadhesive beads of GG:P ionically crosslinked with Al^3+^ ions, achieving a high %EE (around 75%). The system was characterized and evaluated regarding cytotoxicity and in vitro intestinal permeability using a triple co-culture cell model. Beads were circular, with an average diameter of 913 μm. FTIR analysis showed that RES must be physically entrapped within the polymer network. The system was able to reduce drug release in acidic media compared to free RES, releasing only 17.6% in a pH of 1.2, and control drug release for up to 48 h in a pH of 6.8. Release data of RES from the beads correlated better with Weibull’s model and drug transport followed Fickian diffusion and Case II transport mechanisms. Cell viability studies showed that beads did not show toxicity towards a triple co-culture cell model, and even when a discrete cytotoxic effect was observed at the highest concentration evaluated in this work, the viability results were very close to the threshold value of 70%. The encapsulation of RES allowed for a significant reduction in its permeability across a triple co-culture cell model: a favorable feature for the targeted action of RES on colonic cells. In this way, the ionically-crosslinked GG:P beads represent a promising carrier system for the control of drug release throughout the GIT and the targeting of RES to the colon for the treatment of local diseases with the potential to improve the local action of the drug for the treatment of several pathologies in this organ.
